# MicroRNA-130b attenuates dexamethasone-induced increase of lipid accumulation in porcine preadipocytes by suppressing PPAR-γ expression

**DOI:** 10.18632/oncotarget.21318

**Published:** 2017-09-27

**Authors:** Shifeng Pan, Yixin Cui, Xuan Dong, Tangjie Zhang, Hua Xing

**Affiliations:** ^1^ College of Veterinary Medicine, Yangzhou University, Yangzhou, Jiangsu, 225009, P. R. China; ^2^ Jiangsu Co-Innovation Center for the Prevention and Control of Important Animal Infectious Disease and Zoonoses, Yangzhou, Jiangsu, 225009, P. R. China

**Keywords:** miR-130b, PPAR-γ, porcine preadipocytes, lipid accumulation, dexamethasone

## Abstract

In this study, two experiments were conducted to determine the role of miR-130b in dexamethasone (DEX)-induced lipid accumulation. Porcine preadipocytes were treated with 10^−6^ M DEX for 48 h to investigate effects of DEX in lipid accumulation. Next, in order to illustrate the regulatory role of miR-130b on lipid accumulation induced by DEX, miRNA scrambled control (miR-SC), miR-130b overexpression plasmid and miR-130b inhibitor were respectively transfected into porcine preadipocytes at 24 h before DEX treatment for 48 h (miR-SC-DEX, miR-130b-DEX and miR-130b-inhibitor-DEX). Results showed that 10^−6^ M DEX significantly increased TG concentration and expression of miR-130b as well as its target gene peroxisome proliferator-activated receptor-γ (PPAR-γ). Dual-luciferase reporter assays indicated that PPAR-γ expression was negatively regulated by miR-130b, while this effect was abolished with cotransfection of miR-130b and miR-130b inhibitor. In addition, miR-130b-DEX did not change cell proliferation but significantly decreased TG concentration and PPAR-γ expression compared to miR-SC-DEX cells, while miR-130b-inhibitor-DEX cells presented opposite results. Furthermore, miR-130b-DEX significantly reduced expression of PPAR-γ downstream factor perilipin 1 as well as adipogenesis genes fatty acid synthase, acetyl coenzyme A carboxylase, 11β hydroxysteroid dehydrogenase type 1 and fat mass and obesity-associated gene, whereas expression as well as enzyme activity of adipose triglyceride lipase and hormone-sensitive lipase were greatly increased. Overall, these results clarified the role of miR-130b in DEX-induced increase of lipid accumulation in porcine preadipocytes, suggesting that miR-130b might be deemed as a novel potential therapeutic target for DEX-induced increase of lipid accumulation, and consequently provide new insights in obesity control.

## INTRODUCTION

Worldwide obesity epidemic has increased explosively over the last several decades, which rapidly predispose obese individuals to a greater risk for non-alcoholic fatty liver disease, type II diabetes and atherosclerotic cardiovascular diseases [1–3], therefore, a better understanding of molecular mechanisms that contribute to obesity is crucial for its prevention and treatment. Pig (Sus scrofa) is the most powerful domestic animal in fat deposition, furthermore, the pig is a non-primate mammal that closely resembles man in anatomy, physiology and genetics, and can be served as the most desirable animals for studying obesity and metabolic related diseases [4, 5]. In addition, in the animal husbandry and veterinary industry, a reduction of subcutaneous fat deposition is a major goal in the continued improvement of meat animals. As a result, a deeper understanding of fat deposition in porcine adipose tissue facilitates both meat production industry and biomedical research.

Pathophysiologic increase in fat mass with altered adipogenesis and lipolysis, is the direct reason of obesity [6]. This process can be affected by numerous factors, besides genetic and nutritional factors, hormones related to lipid metabolism play important roles in the development of lipid deposition, among which glucocorticoids (GCs) steroids that were released from the adrenal cortex upon activation of the hypothalamic-pituitaryadrenal axis in response to stress [7], are most important. Numerous studies demonstrated that depending on the physiological conditions, exogenous GCs can cause either direct or indirect effects on the lipid metabolism and leads to adipogenic and lipolytic actions in adipose tissues [8–12], suggesting that the pivotal role of these stress hormones in lipid metabolism is much more controversial. Therefore, the exact role of GCs steroids in lipid accumulation of porcine preadipocytes needs to be further described.

Fat deposition in adipose tissue is contributed by both cell number increase through proliferation and cell size increase through lipid accumulation of adipocytes [13, 14], the latter refers to adipogenesis and is highly orchestrated by multiple transcriptional factors, among which peroxisome proliferators-activated receptor-γ (PPAR-γ) is the primary one [15, 16]. PPAR-γ knock-out mice has been shown decreased fat mass [17], whereas overexpression of PPAR-γ show induced adipogenesis [18]. Thus, PPAR-γ is considered as a means of controlling adipose tissue mass, thereby regarding as a rational therapeutic target for the treatment of obesity. However, whether GCs-induced lipid accumulation is associated with changed PPAR-γ expression needs to be further explored, so as to search for the effective therapeutical target against GCs steroids induced obesity.

In addition to the massive understanding of the protein-coding genes in lipid accumulation, miRNAs stand out as a critical contributing factor just in recent decades. They are a class of non-coding, regulatory RNA molecules, 21-24 nucleotides in length and regulate target genes expression by inducing cleavage of mRNAs or via inhibition of protein translation [19]. Therapeutic means altering miRNAs levels are being explored as novel strategies for clinical use in various diseases [20]. Nowadays, accumulating evidence have indicated that miRNAs are abnormally expressed in adipose tissue and act as either promote [21–23] or inhibit adipogenesis genes expression [24–26]. Therefore, it has been proposed that miRNAs are able to serve as novel targets for anti-obesity therapies by affecting the lipid metabolism process.

miR-130b is a novel lipid metabolism related miRNA that has been found to be involved in several biological processes. Overexpression of miR-130b has been shown to impair adipogenesis in the human primary preadipocytes and 3T3-L1 cell lines [27]. In our previous study, we also demonstrated that miR-130b inhibited adipogenesis in Meishan pigs [28], supporting that miR-130b has an important inhibiting effect on lipid metabolism in adipose tissues. In addition, it has been reported that the expression of miR-130b is extremely sensitive to GCs treatment [29], demonstrating the interaction between GCs and miR-130b. However, until now, very little research has been done to determine the effect of miR-130b treatment on the GCs induced lipid accumulation in porcine preadipocytes and to investigate the therapeutic potential of modulating miR-130b level to treat obesity.

Therefore, the aim of the present study was to investigate the effect of DEX on lipid metabolism in porcine preadipocytes, and to further determine the effect of prior transfection with miR-130b on the lipid deposition induced by DEX and to explore the potential mechanisms. These findings may be helpful in improving the pork quality and in searching for treatment strategies of obesity.

## RESULTS

### Effect of 10^−6^ M DEX on the differentiation induction and lipid metabolism related gene expression of porcine preadipocytes

**Figure 1:**
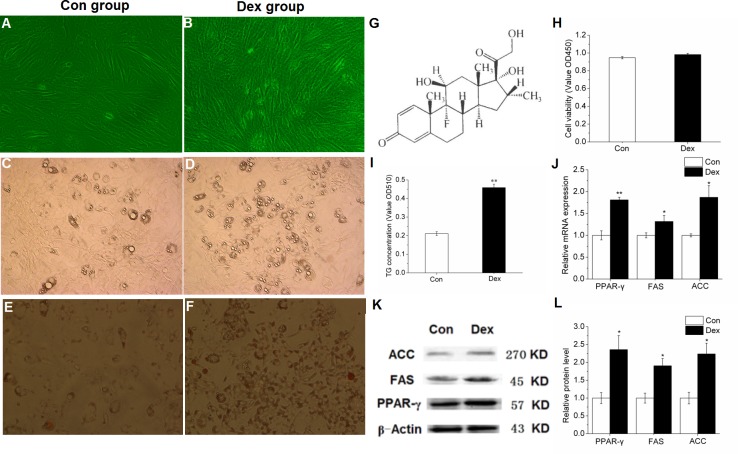
Effect of 10^−6^ M DEX on the differentiation induction and lipid metabolism related gene expression of porcine preadipocytes **(A-B)**: Porcine preadipocytes reached to 85%-90% confluence (×200). **(C-D)**: Lipid droplets under the microscope after differentiation for 48 h (×200). **(E-F)**: Oil Red-O staining with adipocytes, the red parts are lipid droplets (×200). **(G)**: Structural formula of DEX. **(H)**: The cell proliferation and activity. **(I)**: TG quantification. **(J)**: mRNA expression of PPAR-γ, FAS and ACC. **(K-L)**: Protein levels of PPAR-γ, FAS and ACC. Con: control adipocytes. Dex: 10^−6^ M DEX adipocytes. The values shown represent the means±SEM, n=6/group. ^*^P<0.05 *vs.* Con cells, ^**^P<0.01 *vs.* Con cells.

Adipogenic differentiation was induced when the cell density of porcine preadipocytes reached to 85%-90% confluence (Figure 1A-1B), and it can be obviously observed under the microscope that when compared with control cells, the number and the size of lipid droplets in adipocyte increased significantly (Figure 1C-1F) in 10^−6^ M DEX treated cells (Structural formula is shown in Figure 1G), suggesting that the adipogenic differentiation of adipocytes was successfully induced. Furthermore, when compared with control cells, 10^−6^ M DEX did not change the cell viability (Figure 1H), while significantly increased the TG concentration in porcine preadipocytes (Figure 1I) basing on both the Oil red O staining and TG quantitative detection kit results. These above results indicated that 10^−6^ M DEX increased lipid accumulation in porcine preadipocytes, and this was mainly caused by induced differentiation rather than adipocyte proliferation.

In addition, when compared to control cells, 10^−6^ M DEX notably increased PPAR-γ and its target genes FAS and ACC mRNA expression in porcine preadipocytes (Figure 1J). Furthermore, the protein levels of PPAR-γ, FAS and ACC were also significantly increased (Figure 1K-1L), suggesting that PPAR-γ plays an important role in the differentiation induction of porcine preadipocytes under the treatment with 10^−6^ M DEX.

### Effect of 10^−6^ M DEX on the expression of miRNAs targeting PPAR-γ

**Figure 2:**
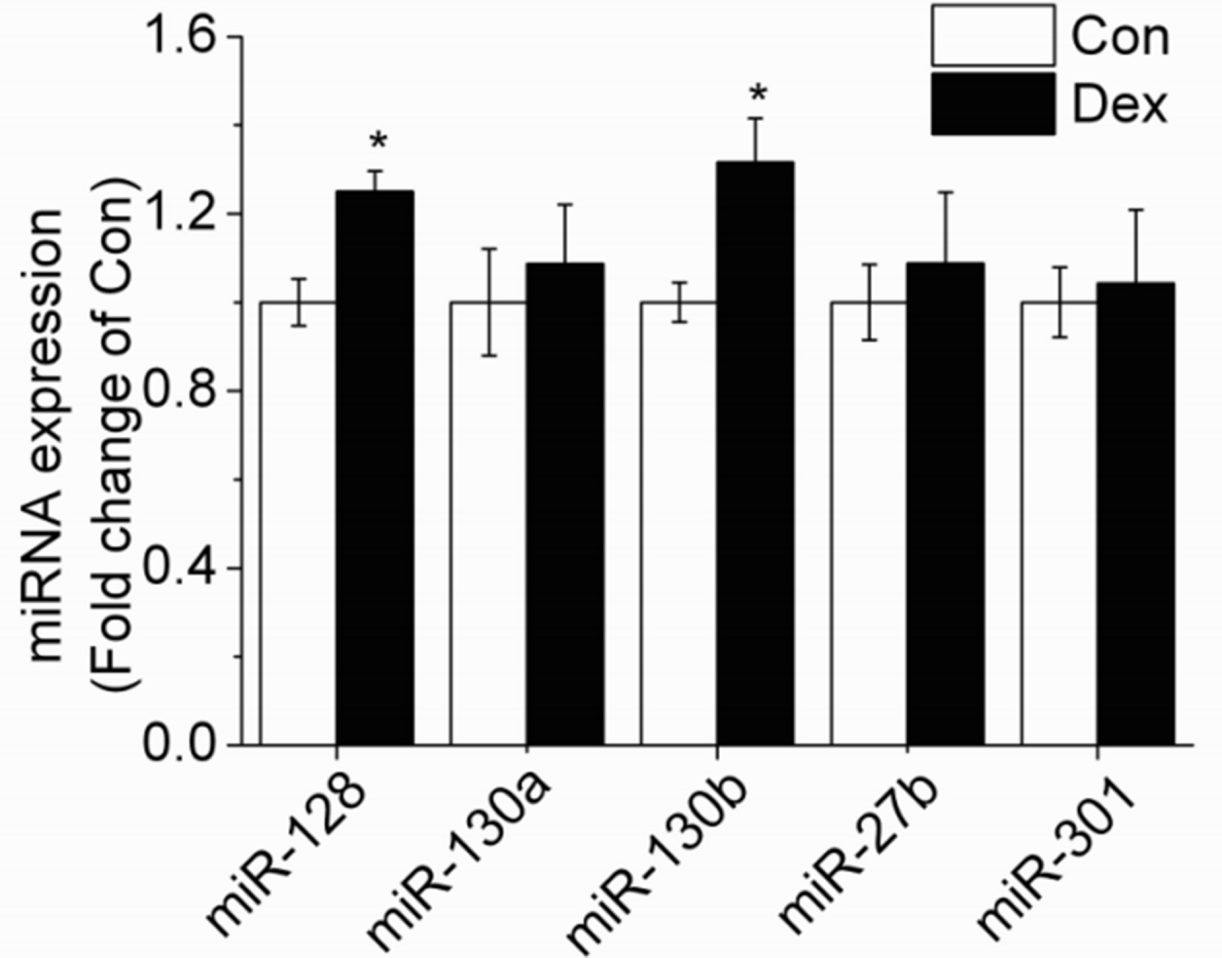
Effect of 10^−6^ M DEX on the expression of miRNAs targeting PPAR-γ in porcine preadipocytes Con: control adipocytes. Dex: 10^−6^ M DEX adipocytes. The values shown represent the means±SEM, n=6/group. ^*^P<0.05 *vs.* Con cells.

Several computational miRNA target-gene prediction tools (http://www.targetscan.org/, http://www.microrna.org, http://www.ebi.ac.uk/enrightsrv/microcosm/) were used to identify possible candidates targeting PPAR-γ. RT-qPCR method was used to detecte the expression of miRNAs and the results showed that only miR-130b and miR-128 expression was significantly increased in 10^−6^ M DEX treated adipocytes when compared with control cells, while miR-130a, miR-27b and miR-301 expression showed no obvious change, indicating that miR-130b expression is sensitive to 10^−6^ M DEX and there may be a certain relationship between miR-130b and PPAR-γ (Figure 2).

### Validation of ssc-miR-130b targeting PPAR-γ 3′-UTR

**Figure 3:**
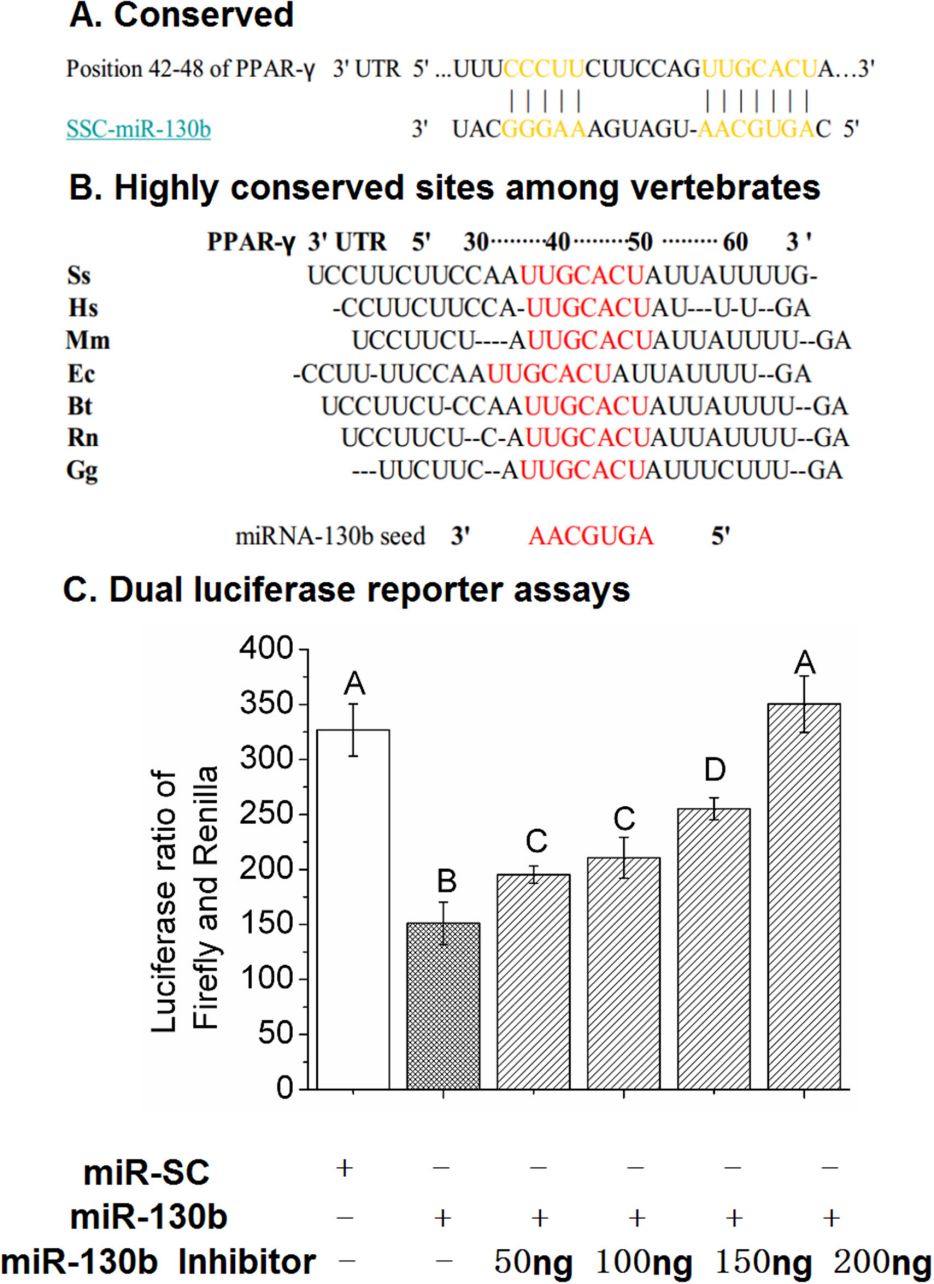
The miR-130b target site in the 3′-UTR of PPAR-γ and dual-luciferase activity assay **(A)**: The predicted, single binding site of miR-130b in the 3′-UTR of pig PPAR-γ. **(B)**: Conservation of the miR-130b binding region in the PPAR-γ 3′-UTR among different species, with the miR-130b seed match highlighted in red (TargetScan). Ss: pig; Hs: human; Mm: mouse; Ec: horse; Bt: cow; Rn: rat; Gg: chicken. **(C)**: Validation of ssc-miR-130b targeting of the PPAR-γ 3′-UTR at 24 h after transfection in HeLa-229 cells. miR-SC: miRNA scrambled control transfected cells. miR-130b: miR-130b transfected cells. miR-130b inhibitor: miR-130b inhibitor transfected cells. The values shown represent the means±SEM, n=6/group. Different letter means significant difference, *P*<0.05.

The 3′-UTR of PPAR-γ mRNA contains the only one predicted miR-130b target site corresponding perfectly to nucleotides 2-8 of mature miRNA (Figure 3A). The seed target sequence is highly conserved in many species including pig, human, mouse, rat, cow, sheep, chicken, and dog (Figure 3B). To examine the relationship between miR-130b and PPAR-γ and confirm that the predicted seed sequence for miR-130b is mediating the repressive effect on PPAR-γ translation, we generated a luciferase reporter DNA construct containing the pig 387 bp PPAR-γ 3′-UTR with a miR-130b putative binding site and pig miR-130b overexpression plasmid (ssc-miR-130b), both of which were cotransfected into HeLa-229 cells that have low levels of endogenous miR-130b, ssc-miR-SC plasmid was used as a negative control. Luciferase activity was decreased significantly in the presence of ectopic expression of miR-130b at 24 h when compared with miR-SC cells, in addition, transfection of miR-130b inhibitor in HeLa-229 cells relieved the repression of the luciferase reporter activity cansed by miR-130b overexpression (Figure 3C). Taken together, these data confirmed the presence of a functional and direct miR-130b target site in the 3′-UTR of PPAR-γ mRNA.

### MiR-130b targets PPAR-γ and regulates lipid metabolism

**Figure 4:**
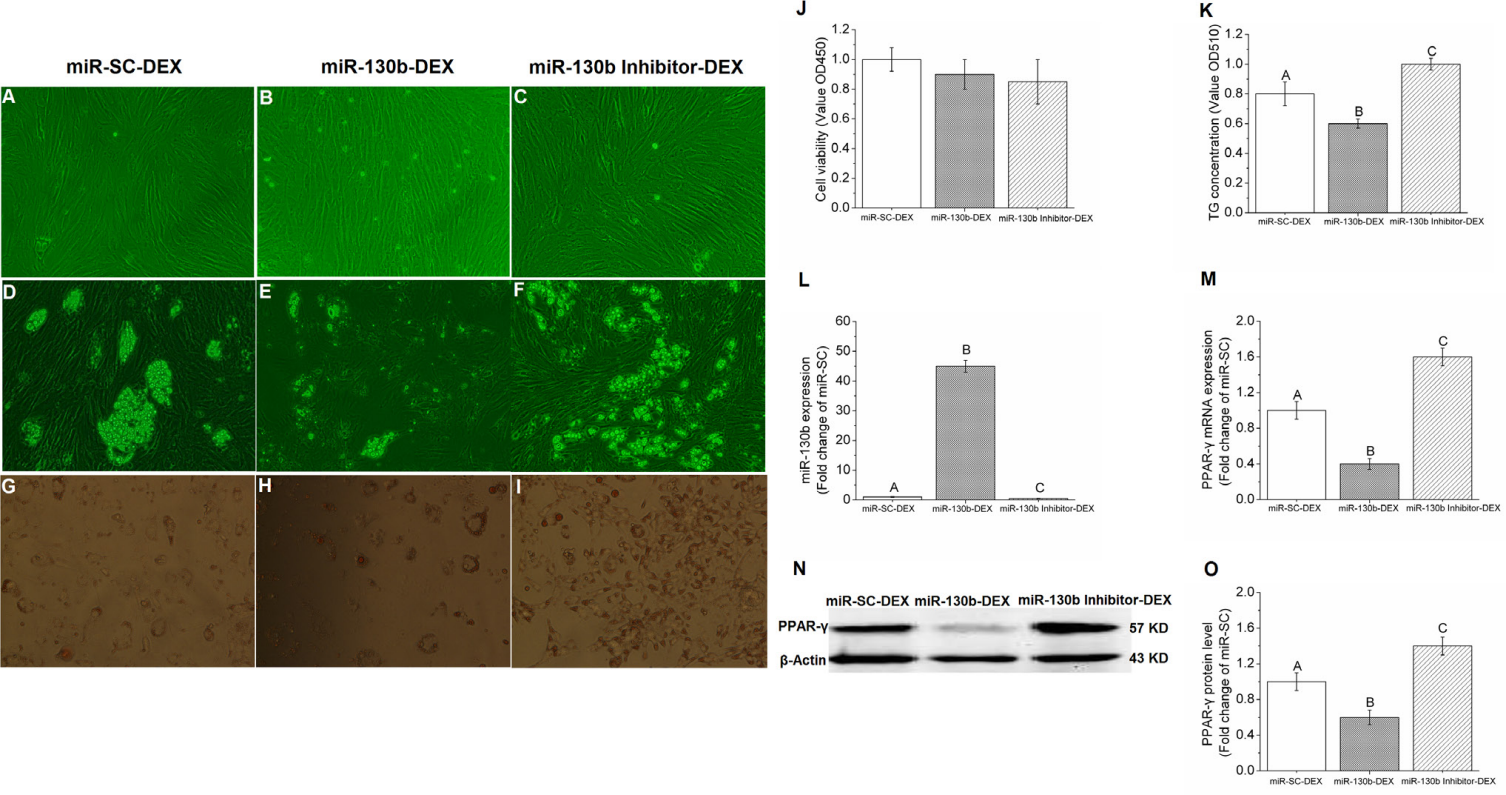
Gain-of-function and loss-of-function of miR-130b in porcine preadipocytes **(A-C)**: Porcine preadipocytes reached to 85%-90% confluence (×200). **(D-F)**: Lipid droplets under the microscope after differentiation for 48 h (×200). **(G-I)**: Oil Red-O staining with adipocytes, the red parts are lipid droplets (×200). **(J)**: The cell proliferation and activity. **(K)**: TG quantification. **(L)**: miR-130b expression. **(M)**: PPAR-γ mRNA expression. **(N)**: Western blot pictures of PPAR-γ. **(O)**: PPAR-γ protein expression. miR-SC-DEX: cells transfected with miRNA scrambled control for 24 h before DEX treatment. miR-130b-DEX: cells transfected with miR-130b for 24 h before DEX treatment. miR-130b-inhibitor-DEX: cells transfected with 100 ng miR-130b inhibitor for 24 h before DEX treatment. The values shown represent the means±SEM, n=6/group. Different letter means significant difference, *P*<0.05.

Based on the relationship between miR-130b and PPAR-γ, it was then important to further validate the role of miR-130b in regulating target gene PPAR-γ by using gain-of-function and loss-of-function experiments. In the present study, gain-of-function and loss-of-function experiments was performed by 100 ng miR-130b overexpression plasmid and miR-130b-inhibitor, respectively. Results showed that when compared with miR-SC-DEX cells, the number and the size of lipid droplets in adipocyte reduced significantly in miR-130b-DEX treated cells, while miR-130b-inhibitor-DEX cells showed the opposite results (Figure 4A-4F). Furthermore, both the Oil red O staining and TG quantitative detection kit results showed that miR-130b-DEX significantly reduced the TG concentration when compared with miR-SC-DEX cells, while miR-130b-inhibitor-DEX significantly increased the TG concentration, though the cell proliferation and activity were not affected among the three groups (Figure 4G-4K). PCR results demonstrated that miR-130b expression in miR-130b-DEX cells was almost forty-five times higher than miR-SC-DEX cells, while miR-130b-inhibitor-DEX cells showed much lower expression of miR-130b (Figure 4L). In addition, higher expression of miR-130b in miR-130b-DEX cells significantly reduced the PPAR-γ mRNA and protein expression when compared with miR-SC-DEX group, while miR-130b-inhibitor-DEX was able to reverse this trend significantly (Figure 4M-4O). These data further suggest that miR-130b is able to target PPAR-γ and represses its expression from another aspect.

### Effect of ssc-miR-130b on mRNA abundance of lipid metabolism-related genes in cultured porcine preadipocytes

**Figure 5:**
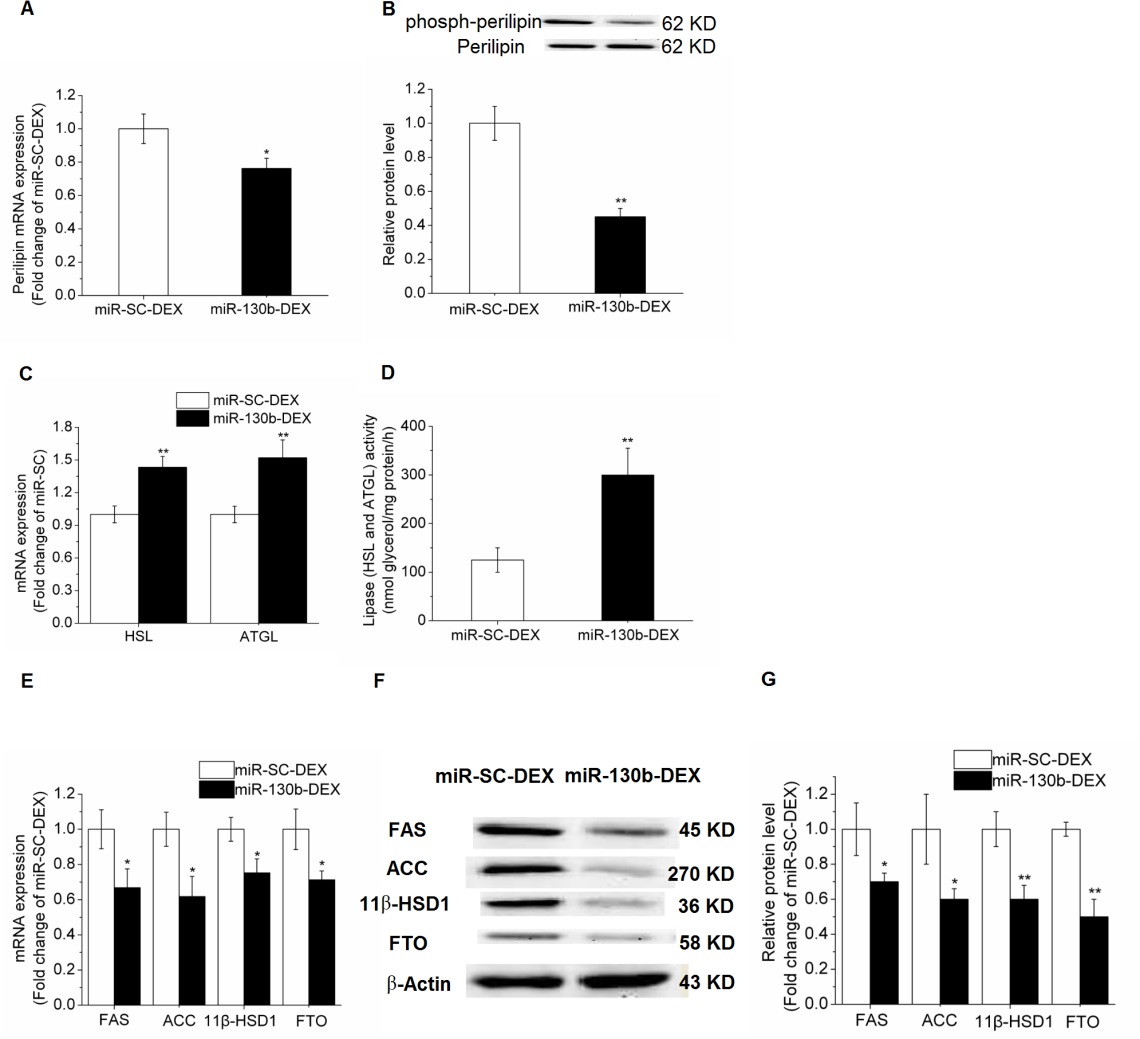
Effect of ssc-miR-130b on mRNA abundance of lipid metabolism-related genes in cultured porcine preadipocytes **(A)**: Perilipin 1 mRNA expression. **(B)**: Phosphorylated protein level of perilipin 1. **(C)**: mRNA expression of HSL and ATGL. **(D)**: Lipase activity of HSL and ATGL. **(E)**: FAS, ACC, 11β-HSD1 and FTO mRNA expression. **(F-G)**: FAS, ACC, 11β-HSD1 and FTO protein levels. miR-SC-DEX: cells transfected with miRNA scrambled control for 24 h before DEX treatment. miR-130b-DEX: cells transfected with miR-130b for 24 h before DEX treatment. The values shown represent the means±SEM, n=6/group. ^*^P<0.05 *vs.* miR-SC-DEX cells.

PPAR-γ is considered as the master regulator of adipogenesis. In the present study, PPAR-γ expression was significantly decreased in the miR-130b transfected cells compared with control cells. mRNA expression and phosphorylated protein level of perilipin 1, a structural lipid droplet protein, which plays a key role in regulating lipolysis, were significantly reduced (*P* < 0.05) in miR-130b-DEX treated adipocytes when compared with miR-SC-DEX cells (Figure 5A-5B). Furthermore, we found that the mRNA expression of adipose triglyceride lipase (ATGL) and hormone-sensitive lipase (HSL) were significantly increased in miR-130b-DEX cells when compared with miR-SC-DEX cells (Figure 5C). In addition, the activity of lipolytic lipases (including HSL and ATGL) in porcine primary cultured adipocytes was significantly higher (*P* < 0.05) in miR-130b transfected adipocytes than that in control adipocytes (Figure 5D).

In addition, qRT-PCR method was performed to examine the effects of the miR-130b on the mRNA abundance of lipid metabolism-related genes in the porcine primary cultured adipocytes. When compared with miR-SC-DEX cells, mRNA expression and protein levels of acetyl-CoA carboxylase (ACC) and fatty acid synthase (FAS) were significantly reduced (*P*<0.05) in miR-130b overexpressed cells. Furthermore, expression of 11beta-hydroxysteroid dehydrogenase type 1 (11β-HSD1) and fat mass and obesity associated gene (FTO), were also significantly depressed (Figure 5E-5G).

## DISCUSSION

Higher levels of lipid accumulation can be due to differentiated adipocytes that undergo hypertrophy (increased size) or hyperplasia (increased numbers due to proliferation) [30], therefore, in the present study we detected both the cell viability and the TG content in the adipocytes. Although the cell viability was not affected after treatment with 10^−6^ M dexamethasone for 48 h, the TG content was significantly increased, indicating that 10^−6^ M dexamethasone enhanced lipid accumulation in porcine preadipocytes mainly depends on increased differentiation and lipid synthesis. GCs have been proposed to have both adipogenic and lipolytic actions within adipose tissue, depending on the concentration, duration, and type of GCs investigated, as well as the experimental model utilized [7, 31–33]. Furthermore, as far as we known, the existing studies focusing on the GCs on lipid metabolism are mainly using cell lines and rodent animals as models, we are the first to study the effect of GCs on fat metabolism in primary cultured porcine adipocytes.

Preadipocytes that gradually filled with lipid droplets and differentiated into mature fat cells were regulated by a number of transcription factors, among which PPAR-γ serves as the primary regulator of lipid metabolism [34]. In the current study, PPAR-γ mRNA and protein expression was significantly increased after treatment with 10^−6^ M dexamethasone for 48 h. This finding is consistent with previous reports demonstrating that PPAR-γ overexpression increases adipogenesis [18]. These results showed that 10^−6^M dexamethasone promotes lipid deposition in primary cultured preadipocytes mainly through up-regulating PPAR-γ expression.

Since PPAR-γ plays an important role in fat metabolism, it is reasonable to reduce fat deposition by regulating the expression of PPAR-γ during the adipocyte differentiation process, eapecially in the exposure of dexamethasone. In recent years, miRNAs were reported to be widely expressed in mammalian adipocytes and played key roles in the process of adipogenesis [35]. More miRNAs need to be digged out to complete the miRNA-gene regulatory modules in porcine adipogenesis. In the present study, we identified seven miRNA candidates targeting PPAR-γ by bioinformatic analyses. Among the predicated miRNAs, only miR-130b and miR-128 were found to be upregulated in the 10^−6^ M DEX treatment cells, demonstrating that miR-130b and miR-128 may participate in the regulation of PPAR-γ expression. In the presnt study, we demonstrated for the first time that miR-130b and miR-128 may be able to affect the differentiation process under the treatment with 10^−6^ M DEX. In general, miRNAs mainly act as negative regulators of target genes, however, here we found miR-128 and miR-130b as well as their target gene PPAR-γ expression were all significantly increased in 10^−6^ M DEX treated adipocytes, suggesting that the increased expression of PPAR-γ may promote the increase of the expression of its potential negative regulators miR-128 and miR-130b by feedback, to maintain self-homeostasis in adipocytes. Indeed, we and others have previously reported that miR-130b was served as a anti-adipogenesis factor in human adipocytes and maternal low protein offspring piglets since miR-130 strongly affected adipocyte differentiation by suppressing PPAR-γ expression [27, 28]. Dual-luciferase reporter assays were performed to confirm direct targeting between miR-130b and PPAR-γ and the results showed that miR-130b overexpression significantly reduced the activity of a luciferase reporter containing the PPAR-γ 3′-UTR after cotransfection for 24 h, while cotransfection with miR-130b inhibitor could significantly reverse this suppression, indicating that miR-130b can directly recognise and bind to the 3′-UTR of PPAR-γ and suppress PPAR-γ expression. It is further elaborated from another aspect that miR-130b was identified as a central modulator of the effects on PPAR-γ inhibition and fat deposition basing on loss- and gain-of-function studies. Although previous study showed that miR-128-3p overexpression was able to effectively inhibit PPAR-γ expression in human hepatic stellate cells [36], in the present study, miR-128 did not alter the activity of the luciferase reporter that has PPAR-γ 3′-UTR (data not shown). Since one miRNA can have a number of target genes, therefore, other potential targets of miR-128 involved in DEX induced lipid accumulation need to be further studied.

In addition, several lines of evidence demonstrate that perilipin 1, a structural lipid droplet protein, is closely associated with the periphery of lipid storage droplets in cultured adipocytes and plays a key role in regulating lipolysis [37, 38]. In the basal state, perilipin 1 is located on the lipid droplet surface of an adipocyte along with other lipid droplet-associated proteins, including ATGL, and provides a barrier function between cellular lipases and their substrates [39, 40]. Upon lipolytic stimulation, phosphorylation of perilipin 1 promotes the release of ATGL coactivator comparative gene identification-58 (CGI-58) [41–43] and recruits HSL to the lipid droplet surface [44, 45] to stimulate the hydrolytic activity of ATGL and facilitate the access of HSL to the lipid substrates, respectively. It has been reported that the promoter sequence of the perilipin 1 gene contains a PPAR-γ response element binding site and that exposing differentiating 3T3-L1 adipocytes to a PPAR-γ agonist significantly augments perilipin 1 mRNA expression [39]. Therefore, the reduced perilipin 1 mRNA expression in the present study may be the consequence of downregulated PPAR-γ expression. It has also been proposed that, reduced perilipin 1 may allow more HSL and ATGL to enter into lipid droplets, and enhance lipolysis in target cells, leading to less lipid accumulation [46].

It is widely recognized that each miRNA has multiple target genes that may function through different pathways [47]. Apart from PPAR-γ, 11beta-hydroxysteroid dehydrogenase type 1 (11β-HSD1) is also predicted to be targeted by miR-130b through bioinformatics methods (data not shown here). Multiple evidence supports that increasing the amount of 11β-HSD1 in the adipose tissue promotes adipogenesis [48], and 11β-HSD1 inhibitor prevents human adipogenesis [49]. In the present study, a significant decrease of 11β-HSD1 mRNA expression was observed after exposure to miR-130b-DEX for 48 h when compared with miR-SC-DEX treated cells. As a matter of fact, excess GCs promote lipid deposition and the main regulator of intracellular GCs levels is 11β-HSD1, which converts inactive GCs into bioactive forms [50]. Inhibition of 11β-HSD1 represents a therapeutic target for treating dexamethasone induced obesity. Inhibition of 11β-HSD1 expression may, therefore, be indicative of suppressed adipogenesis in adipocytes exposed to miR-130b. However, until now, only few studies were focused on the regulatory role of miRNAs in 11β-HSD1 expression, since only miR-561 and −579 are reported to be involved in the regulation of 11β-HSD1 expression [51]. However, in the present study, whether decreased 11β-HSD1 expression was caused by miR-130b overexpression directly or by downregulated PPAR-γ indirectly needs to be further studied, because previous studies demonstrated that PPAR-γ inactivation was able to reduce 11β-HSD1 activity [52, 53]. Furthermore, expression of FTO gene was also significantly reduced, indicating that lower expression of FTO might be related to suppressed lipid deposition in adipocytes. And this is in accordance with previous reports that downregulated FTO expression is associated with less fat deposition in both adipocytes [54] and adipose tissue [55].

We demonstrated in the present study that expression of FAS and ACC, two key enzymes of de novo lipogenesis, was significantly decreased, suggesting that miR-130b inhibited the de novo lipogenesis of porcine adipocyte. This is in line with the known role of PPAR-γ as the master regulator of lipogenesis, driving the expression of numerous of enzymes which are great importances in lipogenesis, including FAS and ACC. In addition, increased expression and the activity of lipolysis enzymes ATGL and HSL suggested activated lipolysis. This is probably contributed by the decreased expression of PPAR-γ, since previous study has showed that down-regulated PPAR-γ can stimulate lipolysis, by up-regulating HSL and ATGL expressions [56]. These above results suggested that reduced fat deposition were caused mainly by both repressing adipogenesis and stimulating lipolysis, demonstrating that miR-130b has important roles in regulating fat deposition. However, targeting miR-130b for therapy may have to be tissue specific and will have to be carefully monitored for undesired side-effects, therefore, a complete understanding of the biological functions of miR-130b in an *in-vivo* model requires further study.

In conclusion, our results showed that 10^−6^M DEX plays a key role in promoting adipogenesis and fat deposition in primary porcine preadipocytes by increasing PPAR-γ expression, furthermore, we demonstrated that this effect can be reversed by prior treatment with miR-130b, probably by the decrease of PPAR-γ and its related gene expression. Further research will be directed at verifying the efficacy of miR-130b in *in vivo* models. These results provide preliminary evidence showing that miR-130b might be useful for treating DEX caused obesity due to their modulatory effects on fat deposition by affecting adipocyte differentiation.

## MATERIALS AND METHODS

### Reagents, cells, and antibodies

Dulbecco's modified Eagle's media: Nutrient Mixture F-12 (DMEM/F-12) was supplied by Life Technologies Inc. (Carlsbad, CA, USA). Fetal bovine serum (FBS) was obtained from HyClone (Logan, UT, USA). The human cervix cancer cell line HeLa-229 was purchased from Cell Bank of Chinese Academy of Sciences (Shanghai, China). Antibodies against the following proteins were used: PPAR-γ (rabbit polyclonal, 1: 500, AP0686, Bioworld Technology, Inc), ACC (rabbit polyclonal, 1: 500, 21923-1-AP, Proteintech^TM^), FAS (rabbit polyclonal, 1: 500, 13098-1-AP, Proteintech^TM^), 11β-HSD1 (rabbit polyclonal, 1: 1000, BS5588, Bioworld Technology, Inc), FTO (rabbit polyclonal, 1: 1000, 14386S, Santa Cruz), and β-actin (mouse monoclonal, 1: 5000, sc-130656, Santa Cruz). Synthetic RNA molecules and scrambled negative control oligonucleotides were purchased from Invitrogen Life Technologies (Carlsbad, CA, USA). 4% paraformaldehyde, 20% glutaraldehyde, pentobarbital sodium, heparin.

### Cell culture

35 day Meishan piglets were killed by exsanguination in a manner approved by the Yangzhou University Institutional Animal Care and Use Committee. Preadipocytes from 6 piglets were isolated according to published protocols [57, 58] with the following modifications and pooled together. Subcutaneous adipose tissue was collected from the neck and back of the piglets and rinsed with serum-free DMEM/F-12 medium supplemented with 15 mM NaHCO_3_, 100 IU/mL penicillin (ST488-1, Beyotime, Shanghai, China) and 100 IU/mL streptomycin (ST488-2, Beyotime, Shanghai, China). The tissue mass was cut with scissors into fine pieces and digested with DMEM/F-12 + 20 g/L BSA (GIBCO) (Invitrogen, Carlsbad, CA, USA) + 1 g/L type IV collagenase (GIBCO) (Invitrogen, Carlsbad, CA, USA) at 37°C in a shaking water bath for approximately 1 h. Then, DMEM/F-12 medium (Invitrogen Life Technologies, Carlsbad, CA, USA) containing 10% foetal bovine serum (FBS) (Invitrogen Life Technologies, Carlsbad, CA, USA) was added to stop digestion. The solution was filtered through sterile nylon meshes (150 μm pore size, 75 μm pore size, 38 μm pore size and 23 μm pore size) to remove undigested tissue. The filtrate was centrifuged at 1000 rpm for 10 min to separate the floating adipocyte cells from the pellet of porcine preadipocytes. The preadipocytes were then incubated with erythrocyte lysis buffer (0.154 M NH_4_Cl, 10 mM KHCO_3_ and 0.1 mM EDTA) at room temperature for 10 min [59], followed by centrifugation at 800 rpm for 5 min. The preadipocytes pellet was washed with DMEM/F-12, centrifuged, and resuspended in plating medium (20% FBS, DMEM/F-12). Finally, the preadipocytes were seeded in culture plates at a density of 3 × 10^5^ cells/cm^2^. The HeLa-229 cell was cultured in DMEM (Carlsbad, CA, USA) (High glucose) supplement with 10% fetal bovine serum. Both of the cells were incubated at 37°C in a humidified incubator with an atmosphere of 5% CO_2_. The medium was changed every second day.

### Preadipocytes grouping and adipogenic differentiation

In the present study, two experiments were constructed to determine the effect of miR-130b on DEX-induced lipid accumulation and to clarify its potential mechanisms. Cultured porcine preadipocytes were maintained in plating medium until 85 ~ 90% confluence. In the first experiment, cells were randomly divided into two groups: one is control group (Con), the other one is DEX treatment group (DEX), then to induce differentiation, cells were exposed to medium (without FBS) containing ITS (5 U/mL insulin, 5 μg/mL transferrin and 5 ng/mL selenium, Sigma), 400 μM oleic acid (Sigma, St. Louis, MO, USA), and BSA (Invitrogen, Carlsbad, CA, USA) for 48 h, with a ratio of oleic acid to BSA of 6:1. In the second experiment, cells were randomly divided into three groups and transfected with different substances at 24 hours before DEX treatment: miR-130b overexpression plasmid group (miR-130b-DEX), miR-130b inhibitor group (miR-130b-inhibitor-DEX) and miRNA scrambled control group (miR-SC-DEX), then cells were treated with DEX and differentiation medium for 48 h. All preadipocytes were cultured in DMEM/F-12 plus L-glutamine, penicillin (100 IU/mL), streptomycin (100 IU/mL) and fungizone (4 μg/ml) (RS0009, Yocon, Beijing, China), at 37°C with 5% CO_2_.

### Cell proliferation detected by cell counting kit-8 (CCK-8)

For cell proliferation studies, preadipocytes were seeded in 96-well culture plates at a density of 10^3^/well, and then, 100 μL DMEM/F-12 medium containing 10% FBS was added to each well. After 10^−6^ M DEX treatment for 48 h, we test the cell Proliferation with adding 10 μL CCK-8 (Keygentec, Nanjing, China) reagent to each well and then cultured in an incubator for an additional 2 h at 37°C in a humidified, 5% CO_2_ atmosphere. The optical density (OD) was read at 450 nm absorbance on a microplate reader (Synergy^TM^ HTX, BioTek, Winooski, VT, USA).

### TG content determination

The intracellular TG content was measured according to the method of Oil Red O staining extraction. Preadipocytes were seeded in 24-well culture plates until 85~90% confluence, after treatment with 10^−6^ M DEX for 48 h, the old culture medium was removed. Cells were first washed with phosphate buffered saline (PBS) for three times, fixed with 10% formalin for 5 min and changed with fixative for 2 h. After fixation, 60% isopropanol (1 mL/well) was added to the plate for 30 s. After removing 60% isopropanol, Oil Red O working solution (Sunshinebio, Nanjing, China) was added to the plate for 1 h (1 mL/well). And then, Oil Red O working solution was removed and 300 μL 100% isopropanol was added to the plate to extract Oil Red O. Finally, 100% isopropanol was collected and the plate was observed with microscope. A wavelength of 510 nm absorbance value should be determinated with Microplate reader (Synergy BioTek, Vermont, USA), which can reflect the intracellular TG content.

### Lipase (including HSL and ATGL) activity assay

10^7^ adipocytes in 1 mL of homogenization buffer (0.1 mmol/L K^+^-PBS containing 1 mmol/L MgCl2, 1 mmol/L DTT and 1 mmol/L EDTA) was homogenized in ice for 30 min, and then centrifuged at 12,000 × g at 4°C for 10 min. The protein concentration of supernatants was measured with the BCA Protein Assay Kit (Pierce Chemical Corp., Rockford, USA). Triolein without glycerin was used as substrate which can be hydrolysed to glycerol by the two enzymes, HSL and ATGL. The supernatant together with prepared triolein were incubated for 1 h at 37°C. The lipases in supernatant activate the lypolytic degradation of the triolein emulsion. The released glycerol was determined using a commercial kit (Applygen, China). To fit the activities of the enzymes to the linear range of standard curves constructed with pure enzymes, all samples were measured in duplicate at appropriate dilutions. The activity of lipases is expressed as nanomoles released glycerol per milligram protein per hour.

### RNA extraction and real-time PCR

Total RNA was extracted from homogenised adipose cells using the TRIzol Total RNA Kit (Invitrogen Life Technologies, Carlsbad, CA, USA) according to the manufacturer's instructions and reverse-transcribed with the PrimeScript First Strand cDNA Synthesis kit (no. D6110A, Takara). Two microliters of diluted cDNA (1: 20) were used in each real-time PCR assay by Mx3000P (Stratagene). Peptidylprolyl isomerase A (PPIA) was chosen as a housekeeping gene after comparing with GAPDH and β-actin through NormFinder analysis, because it is expressed in abundance comparable to the genes of interest and its expression was not affected by the treatment. Relative mRNA levels were determined by comparing the PCR cycle threshold between control and 10^−6^ M DEX groups after normalizing for PPIA. Data are shown as fold change compared to the control group. All primers were synthesized by Genewiz, Inc (Suzhou, China) and listed in Table 1.

**Table 1:** Primers used in the present study

Name	Sequence
Plasmids construction	
*ssc-miR-130b (MI0013136)*	F: GATCCGCCTGCCTGACACTCTTTCCCTGTTGCACTACTGTGGGCCACTGGGAAGCAGT GCAATGATGAAAGGGCATCAGTCAGGCTTTTTTGGAAA
	R: AGCTTTTCCAAAAAAGCCTGACTGATGCCCTTTCATCATTGCACTGCTTCCCAGTGGC CCACAGTAGTGCAACAGGGAAAGAGTGTCAGGCAGGCG
*ssc-miR-SC*	F: GATCCGACTTACAGCCAGTTCCTAGTATAGTGAAGCAGCAGATGGTATACTAGGAAC TGGCTGTAAGCTTTTTTTGGAAA
	R: AGCTTTTCCAAAAAAAGCTTACAGCCAGTTCCTAGTATACCATCTGCTGCTTCACTAT ACTAGGAACTGGCTGTAAGTCG
mRNA expression	
*PPAR-γ* (NM_138711)	F: GCCCTTCACCACTGTTGATT
	R: GAGTTGGAAGGCTCTTCGTG
*FAS* (EF589048)	F: GTCCTGCTGAAGCCTAACTC
	R: TCCTTGGAACCGTCTGTG
*ACC* (NM_133360.2)	F: GGCCATCAAGGACTTCAACC
	R: ACGATGTAAGCGCCGAACTT
*11β-HSD1* (AF414124)	F: CCATGCTGAAGCAGAGCAAC
	R: AAGAACCCGTCCAGAGCAAA
*FTO* (FJ853994)	F: GGAGAAAGCCAATATCGACACC
	R: TCTGCTCTTCCTGTCCACCTC
*Perilipin* (AY973170)	F: GCCTGACTTTGCTGGATGG
	R: CTTGGTGCTGGTGTAGGTCTTCT
*HSL* (AY686758)	F: ACCCTCGGCTGTCAACTTCTT
	R: TCCTCCTTGGTGCTAATCTCGT
*ATGL* (EF583921)	F: ACCTGTCCAACCTGCTGC
	R: GCCTGTCTGCTCCTTTATCCA
*PPIA* (NM_214353.1)	F: TCCTCCTTGGTGCTAATCTCGT
	R: TGATCTTCTTGCTGGTCTT

### Bioinformatics method

The miRNA targets predicted by computer-aided algorithms were obtained from miRGen (http://www.diana.pcbi.upenn.edu/miRGen.html) [47], TargetScan (http://www.targetscan.org/vert _42/) [60], PicTar (http://pictar.org/) [61], and miRanda (http://www.microrna.org/microrna/).

### miRNA real-time PCR quantification

RT-PCR analysis of miRNA expression was performed in an Mx3000P (Stratagene, Santa Clara, CA, USA) with specific primers (Table 2). Briefly, total RNA was extracted from adipocytes using TRIZOL Reagent (Invitrogen Life Technologies, Carlsbad, CA, USA) and subsequently purified with the RNase-Free DNase Set (Promega, Madison, USA) according to the manufacturer's instructions. The treated total RNA (4 μg) was polyadenylated by poly(A) polymerase (PAP) at 37°C for 1 h in a 20 μL reaction mixture following the manufacturer's directions for the Poly(A) Tailing Kit (AM1350, Ambion, USA) [61]. The tailing reactions contained 4 μg of RNA samples (1 μg/μL), 4 μL of 5× E-PAP buffer, 2 μL of 25 mM MnCl_2_, 2 μL of 10 mM ATP, 0.8 μL of E-PAP, and the external controls (E1, E2 and E5) at 0.2 pmol each, this reaction solution was brought up to a 20 μL final volume with nuclease-free water. After phenol-chloroform extraction and ethanol precipitation, the RNAs were dissolved in DEPC-treated water and cDNAs were synthesised from tailing RNAs using a gene-specific oligo dT-adapter primer (1 μg/μL). Reverse transcriptase reactions contained 2 μg poly-A tailed RNAs and 1μL of oligo dT-adapter (1 μg/μL). The 10 μL reactions were incubated for 5 min at 70°C (RT1). The RT2 reactions consisted of the entire RT1 reactions, mixed with 5 μL M-MLV 5× buffer (containing 250 mM pH 8.3 Tris-HCl, 15 mM MgCl_2_, 375 mM KCl and 50 mM DTT), 1.25 μL 10 mM dNTP, 1 μL M-MLV RNase (200 U/μL), 0.5 μL RNase inhibitor (40 U/μL). The 25 μL reactions were incubated at 42°C for 1 h and then at 95°C for 5 min. The 25 μL PCR reactions included 2 μL RT product, 2 μL primers (Table 2), 8.5 μL sterile 3d H_2_O, and 12.5 μL SYBR Premix Ex Taq TM (TaKaRa, Tokyo, Japan). The reactions were incubated in a 96-well optical plate at 95°C for 5 min, followed by 28 cycles at 95°C for 30 s and 66°C for 30 s. The PCR reactions run on an Mx 3000P (Agilent Technologies Stratagene, Santa Clara, CA, USA) and analysed using the Mx 3000P System SDS software.

**Table 2:** PCR primers used for mature miRNAs expression in the present study

Name	Sequence
miRNA expression	
*miR-130b* (MIMAT0013922)	F: CAGTGCAATGATGAAAGGGCAT
	R: TAGAGTGAGTGTAGCGAGCA
*miR-130a* (MIMAT0007758)	F: CAGTGCAATGTTAAAAGGGCAT
	R: TAGAGTGAGTGTAGCGAGCA
*miR-27b* (MIMAT0013889)	F: AGAGCTTAGCTGATTGGTGAACA
*miR-128* (MIMAT0002157)	R: TAGAGTGAGTGTAGCGAGCA
	F: TCACAGTGAACCGGTCTCTTT
	R: TAGAGTGAGTGTAGCGAGCA
*miR-301* (MIMAT0002138)	F: CAGTCCAATAGTATTGTCAAAGC
	R: TAGAGTGAGTGTAGCGAGCA
Reverse transcription	
Exogenous reference gene	F: GTGACCCACGATGTGTATTCG
	R: TAGAGTGAGTGTAGCGAGCA
Reverse transcription primer	TAGAGTGAGTGTAGCGAGCACAGCATTAATACGACTCACTATAGG(T)16VN

E5 small nuclear RNA was used as an external control to normalise RNA input. The Ct value is defined as the fractional cycle number at which the fluorescence passes the fixed threshold. The fold change was calculated using the 2^−ΔΔCt^ method, presented as the fold-expression change in DEX-treated adipocytes relative to their corresponding control adipocytes after normalisation to the endogenous control. All experiments were performed in triplicate.

### Determination of protein expression

One bottle (25 cm^2^) of frozen adipocytes was extracted in 1 ml lysis buffer containing 100 mM NaCl, 2 mM EDTA, 5% SDS, 0.1 mM Na_3_VO4, 50 mM NaF, 1 mM benzamidine, 100 μM AEBSF, 10 μg/ml aprotinin and 50 mM HEPES (pH 7.4). The protein content was measured with the BCA Protein Assay Kit (Pierce biotechnology, Rockford, Illinois, USA, 23227). Forty micrograms of protein extract was mixed with loading buffer and denatured by boiling for 5 min before being loaded on a 10% SDS-polyacrylamide gel. After electrophoresis, the proteins were transferred to nitrocellulose membranes and blocked with 3% BSA in Tween-Tris-buffered saline for 90 min at room temperature. After repeated washing with Tween-Tris-buffered saline, the membranes were incubated with the appropriate antibodies. Western blot analysis for detecting PPAR-γ, FAS, ACC, 11β-HSD1 and FTO were performed with different polyclonal antibodies. An antibody against β-actin (Abcam, Cambridge, UK) was used as an internal standard at a 1: 5,000 dilution. Goat anti-rabbit IgG peroxidase-conjugated secondary antibodies (Bioworld Technology, Minneapolis, USA) were used at a dilution of 1: 10,000. Finally, the bands were visualized by enhanced chemiluminescence with the LumiGlo substrate (Super Signal West Pico Trial Kit, Pierce, Rockford, IL, U.S.A.) and captured by VersaDoc 4000MP system (Bio-Rad, Hercules, CA, U.S.A.) to calculate the value of band density using Quantity One software (Bio-Rad) automatically. The protein content was presented as the fold change relative to the control. The experiments were performed in triplicate.

### Plasmid construction

A 387 bp fragment of the PPAR-γ 3′-UTR, which contains one predicted conserved target site of miR-130b, was amplified by PCR using the primers 5′-GCTGCTGCAAGTAATAAAG-3′ and 5′-TAAAGGAAGGAAGAGGGAG-3′. The 387 bp fragment, which contains a motif for miR-130b that is broadly conserved in vertebrates (www.targetscan.org), was subcloned into the luciferase reporter pGL3-Control using XbaI (Invitrogen Life Technologies, Carlsbad, CA, USA). These constructs were named pGL3-control/ PPAR-γ.

Approximately 89 bp precursors of miR-130b and miR-SC were synthesized by Invitrogen Life Technology (Carlsbad, CA, USA). The synthesis was based on the sequence information from pig miRNA precursors (www.mirbase.org) and the requirements for p*Silencer* 3.1-H1 siRNA expression vector (Ambion, Austin, TX, USA). Precursors of miR-130b and miR-SC were annealed using annealing buffer (5×), miRNA precursor upstream sequence (50 μmol/L) and downstream sequence (50 μmol/L) (Table 1). The 50 μL solutions were incubated in 96-well optical plates at 95°C for 2 min, and subjected to touchdown PCR. During this procedure the temperature was decreased 0.1°C every 8 s until it reached 25°C. The PCR products were subcloned into pSilencer 3.1-H1 siRNA expression vector using BamHI and HindIII restricted endonuclease (Invitrogen Life Technologies, Carlsbad, CA, USA).

### DNA transfection

Approximately 3×10^4^/cm^3^ HeLa cells were seeded and cultured in 25 cm^2^ cell culture bottles. When the cells reached 90-95% confluence, they were cotransfected with 100 ng of pGL3-control/ PPAR-γ 3′-UTR fluorescent luciferase reporter plasmid, 10 ng of pRL-TK plasmid (used to normalise for transformation efficiency), or 100 ng of pSilence 3.1 H1-neo miR-130b with Lipofectamine 2000 (Invitrogen Life Technologies, Carlsbad, CA, USA) according to the manufacturer's instructions. Negative controls were cotransfected with 100 ng of miR-SC, 100 ng of pGL3-control/ PPAR-γ 3′-UTR fluorescent luciferase reporter plasmid and 10 ng of pRL-TK plasmid. At the same time, different concentrations of miR-130b inhibitor (Invitrogen Life Technologies, Carlsbad, CA, USA) were added to the medium of the cotransfected cells. The transfected HeLa cells were incubated at 5% CO_2_ and 37°C for 24 h.

### Dual luciferase activity assay

Twenty-four hours after transfection, firefly and renilla luciferase activities were measured using a Dual-Luciferase Assay Kit (Promega, Madison, USA) with a plate reader (Perkin Elmer, Waltham, MA, USA). The renilla and firefly luciferase signals were detected using the Veritas Microplate Luminometer (Turner Biosystems, Sunnyvale, CA, USA). The firefly luciferase signal was normalized to the renilla luciferase signal. The normalized firefly luciferase activity was compared between miR-130b and miR-SC cells. The results were expressed as relative activity. Each target construct was tested in triplicate, and the assay was repeated to confirm the results.

### Statistical analysis

All data are presented as the mean ± SEM. Statistical analyses were carried out with Statistical Program for Social Sciences (SPSS) software 20.0 for Windows (SPSS Inc., Chicago, IL, USA). The differences were tested with a one-way ANOVA. A *P*-value of less than 0.05 was considered significant.

## Abbreviations

ACC: acetyl coenzyme A carboxylase
ATGL: adipose triglyceride lipase
DEX: dexamethasone
DMEM/F-12: dulbecco's modified eagle's media: nutrient mixture F-12
FAS: fatty acid synthetase
FBS: fetal bovine serum
FTO: fat mass and obesity associated gene
HSL: hormone-sensitive lipase
Mir: microRNA
PPAR-γ: peroxisome proliferator-activated receptor-γ
RT-PCR: quantitative real-time polymerse chain reaction
SCD-1: stearoyl-coenzyme A desaturase 1
SPSS: statistical program for social sciences
TG: triglycerides
3′-UTR: 3′-untranslated region
11β-HSD1: 11β hydroxysteroid dehydrogenase type 1
